# Aerodynamic Super-Repellent Surfaces

**DOI:** 10.34133/research.0111

**Published:** 2023-04-19

**Authors:** Fanfei Yu, Jinlong Yang, Ran Tao, Yao Tan, Jinpei Wang, Dehui Wang, Longquan Chen, Zuankai Wang, Xu Deng

**Affiliations:** ^1^ Institute of Fundamental and Frontier Sciences, University of Electronic Science and Technology of China, Chengdu 610054, P. R. China.; ^2^ Department of Mechanical Engineering, City University of Hong Kong, Kowloon, Hong Kong Special Administrative Region 999077, P. R. China.; ^3^ School of Physics, University of Electronic Science and Technology of China, Chengdu 610054, P. R. China.; ^4^ Department of Mechanical Engineering, Hong Kong Polytechnic University, Kowloon, Hong Kong Special Administrative Region 999077, P. R. China.; ^5^ Shenzhen Institute for Advanced Study, University of Electronic Science and Technology of China, Shenzhen 518110, P. R. China.

## Abstract

Repelling liquid drops from engineering surfaces has attracted great attention in a variety of applications. To achieve efficient liquid shedding, delicate surface textures are often introduced to sustain air pockets at the liquid–solid interface. However, those surfaces are prone to suffer from mechanical failure, which may bring reliability issues and thus limits their applications. Here, inspired by the aerodynamic Leidenfrost effect, we present that impacting drops are directionally repelled from smooth surfaces supplied with an exogenous air layer. Our theoretical analysis reveals that the synchronized nonwetting and oblique bouncing behavior is attributed to the aerodynamic force arising from the air layer. The versatility and practicability of our approach allow for drop repellency without the aid of any surface wettability treatment and also avoid the consideration of mechanical stability issues, which thereby provides a promising candidate for the applications that necessitate liquid shedding, e.g., resolve the problem of tiny raindrop adhesion on the automobile side window during driving.

## Introduction

**Fig. 1.**
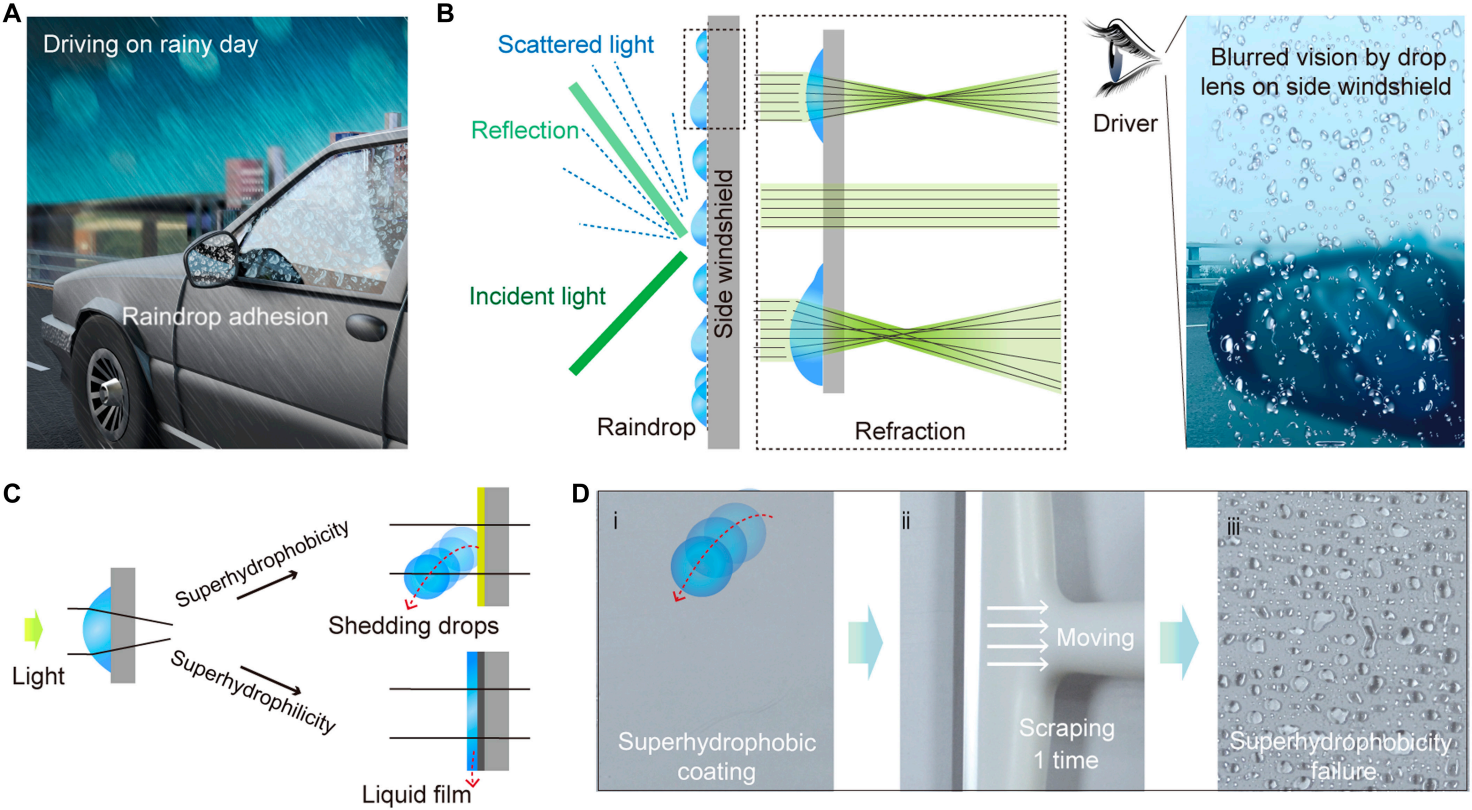
Blurred vision caused by drop adhesion. (A) A photo shows the blurred vision caused by raindrop adhesion. (B) Effect of surface raindrop adhesion on light scattering, reflection, and refraction. (C) Surface modification to resolve the problem of raindrop adhesion. (D) The poor mechanical stability of the micro/nanostructured superhydrophobic coating.

Traffic safety threatens the lives of humankind. Every minute, more than 2 people die because of traffic [1]. Notably, the traffic accident rate on rainy days is much higher than on sunny days [2–4], for which an important factor is the obstruction of vision caused by raindrop adhesion. Generally, raindrops on the front windshield or rearview mirror can be eliminated by wipers operating or heating, while raindrop adhesion on the side windshield, which may also cause undesirable consequences, is still an unsolved issue. The adhering raindrop on the side windshield acts like a convex lens, which scatters lights and thus distorts the visual scene image (Fig 1A), as the scene of blurred vision we simulate in Fig. 1B. This greatly worsens the drivers’ horizons and may lead to catastrophic car accidents [5–8]. Therefore, a strategy to efficiently remove the adhered drops and eliminate blurred vision is expected.

To address the issue of blurry side windshield, surface modification might be considered at first thought. By rendering the surface superhydrophobicity or superhydrophilicity [9–18], the solid–liquid interaction could be alleviated or reinforced and thus facilitates rapid liquid drop shedding or forms liquid films to avoid the optical effects of drop curvature (Fig. 1C). However, commercial superwetting materials often suffer from durability issues due to the fragility of their encompassing micro/nanostructures, especially in dynamic working conditions (e.g., reciprocating sliding) [19–25]. This is evidenced by Fig. 1D and Fig. S1, where the stability of a glass with a commercial superhydrophobic coating is tested with a glass scraper (mimicking the action of rolling up or down the side windshield). Specifically, the original superhydrophobic-treated area is out of function for water repellency after being scraped once. In addition, the introduction of micro/nanostructures inevitably leads to light scattering, which deteriorates light transmission through surfaces. Therefore, surface modification shows limited performance in dynamic working conditions due to its poor mechanical stability and transparency, although they may work well at initial use.

Apart from providing air pockets by introducing a physical structure, an alternative strategy for obtaining the nonwetting surface property is to generate an air cushion at the solid–liquid interface, which is capable of preventing the incoming liquid drops from contacting the surface fundamentally [26–28]. The well-known Leidenfrost effect induced by overheating enables volatile liquids to levitate above a vapor cushion [29–32]. More intriguingly, the dynamical motion of surfaces (over a threshold velocity) can also engender an air layer to repel the incoming drop, known as the aerodynamic Leidenfrost effect [33–36]. Unfortunately, these methods necessitate external actuation, which makes the experimental setup complex and cumbersome and may bring reliability issues.

Inspired by the aerodynamic Leidenfrost effect, in this work, we demonstrated that incoming drops are repelled and directionally removed by glass surfaces supplied with an exogenous air layer. The threshold speed of drop bouncing, in particular as a function of the drop impact velocity, is explored experimentally. Theoretical analysis discloses that the synergic effect of nonwetting and oblique bouncing is induced by the aerodynamic force arising from the air layer. Such a strategy can effectively realize drop repellency avoiding the surface inherent wettability and mechanical stability, providing a promising candidate for resolving the problem of raindrop adhesion on the automobile side windshield.

## Results

### Introduction of an exogenous air layer

**Fig. 2.**
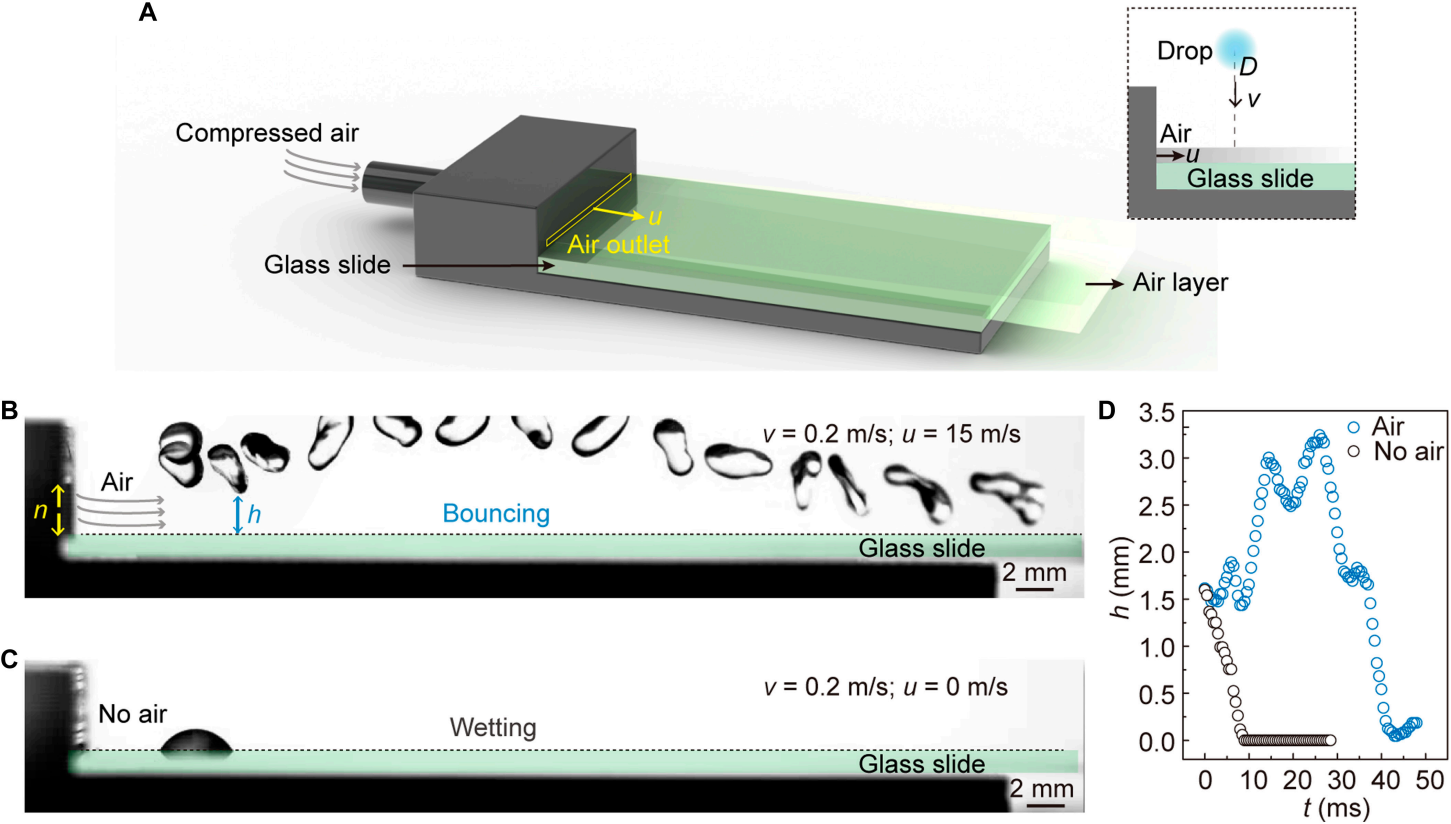
Aerodynamic repellency of impacting drop by introducing an air layer. (A) Design of air layer introduction device. The inset shows the side-view schematic diagram of a drop impacting the air layer surface. (B) Impacting drop bounced off an air layer glass surface. The impacting velocity *v* = 0.2 m/s, the airflow velocity *u* is 15 m/s, the thickness (*n*) of the air outlet is 3 mm, and the diameter of the impacting drop *D* is 2.5 mm. (C) Impacting drop wetted the glass surface at a velocity of 0.2 m/s without introducing an air layer. (D) The height (*h*) change curves of the lowest part of the impacting drop from the surface vs. time (*t*) during the whole impacting process.

An air compressor was used to introduce an air layer onto a glass surface through an air introduction device (Fig. 2A and Fig. S2). The device consisted of a round air inlet and a rectangular air outlet with adjustable thickness. The air inlet was easily connected to the air compressor via a rubber pipe, while the air outlet’s length and width (*m* and *n*, respectively) were optimized for even airflow distribution. The device's internal structure is shown in Fig. S2, where the transitional design between the air inlet and outlet benefits the stabilization of the airflow. The airflow rate (*Q*) was measured by a digital display gas flowmeter (range from 0 to 200 l/min). The initial flow velocity of the air layer was determined by the size of *m* and *n*. Using a 3-dimensional (3D) printer, 4 different outlet thicknesses (*n* = 0.6, 1, 2, and 3 mm) were printed with a fixed length *m* = 15 mm.

### Aerodynamic repellency of impacting drop on air layer surface

To investigate drop-impacting dynamics on the air layer surface, an experimental device was set up as shown in the inset of Fig. 2A. Before the drop-impacting experiment, the air compressor was utilized to ensure stable airflow through the glass surface. A drop with a diameter of *D* = 2.5 mm was then dropped onto the air layer surface at an initial velocity of *v* = 0.2 m/s. Subsequently, the drop rebounded from the surface and finally left the glass surface with a length of 75 mm due to the action of the air layer (*Q* = 40.5 l/min, *n* = 3 mm, airflow velocity *u* = *Q*/*mn* = 15 m/s), realizing the nonwetting of glass surface (Fig. 2B and Movie S1). In contrast, once the air layer was stopped (*Q* = 0 l/min, *u* = 0 m/s), the drop deposited on the surface after spreading, retracting, and oscillating, finally wetting the surface (Fig. 2C and Movie S2). It should be noted that impacting position was set 5 mm away from the air outlet unless otherwise specified in subsequent experiments and discussions. To quantitatively describe the dynamic difference between the 2 impacting events, the changes in the distance between the drop and the surface (*h*) with time were monitored. As shown in Fig. 2D, the presence of the air layer ensures that the impacting drop always keeps a distance from the air layer surface without contact, thus realizing nonwetting, in contrast to directly wetting the surface.

**Fig. 3.**
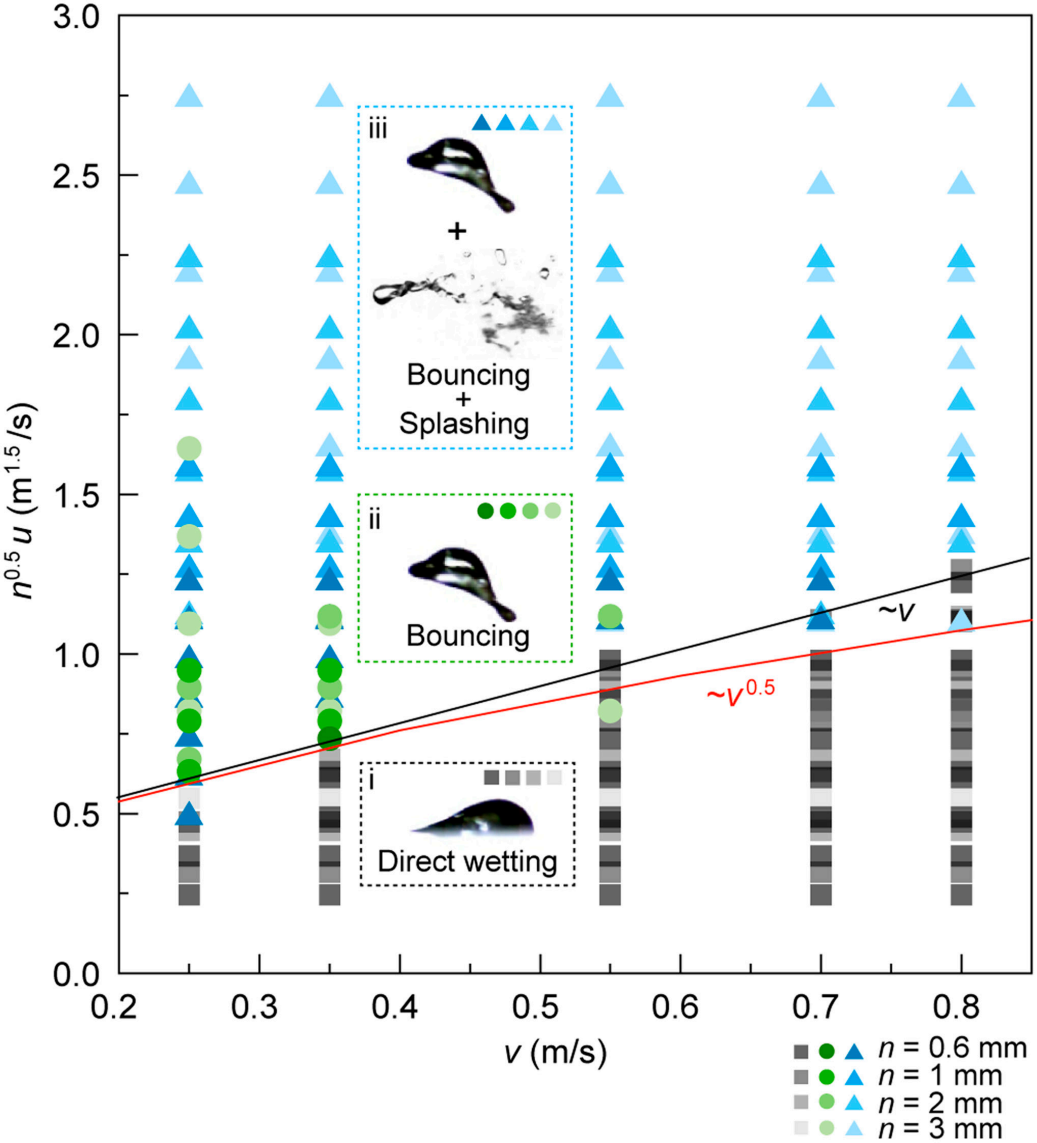
Phase diagram of the impacting outcomes. The outcomes of the drop impacting the air layer surface depend on the energy balance between the drop itself and the air layer. The transition is discontinuous: either the drop wets the surface or it rebounds off the surface and splashes. The red and black curves follow the scaling behavior of *v*^0.5^ and *v* depicted in Eqs. 4 and 6, respectively.

The repulsion of the impinging drop on the air layer surface is conditional and can occur only under certain conditions. Specifically, the appropriate flow rate of the air layer must be maintained. The results showed that a low flow velocity (*u* = 10 m/s) leads to a weak air layer that was unable to resist the impact energy of the drop, resulting in direct wetting (Fig. S3A and Movie S3). Conversely, a high flow velocity (*u* = 25 m/s) causes the impacting drop to rapidly ricocheted off the surface while also deforming and splashing due to the strong aerodynamic force of the air layer (Fig. S3B and Movie S4), resulting in a mixed mode that includes drop bouncing and splashing. The repellency threshold u* is determined not only by the critical *u* when the releasing drops impacting the air layer surface at a fixed velocity get stuck but also by the thickness of the air layer modified by the air outlet thickness *n*. The experiment further explored the relationship between drop impact velocity *v*, airflow velocity *u*, and air layer thickness *n* and their effect on drop dynamics. The results show that the transition between different modes of drop behavior is discontinuous: either the drop wets the surface, or it fully bounces and splashes (Fig. 3 and Fig. S3C to F). The phase diagram suggested that the impact energy (*v*) and the energy provided by the air layer (*u*, *n*) jointly determine the impacting modes.

### Mechanism of drop repulsion on air layer surface

**Fig. 4.**
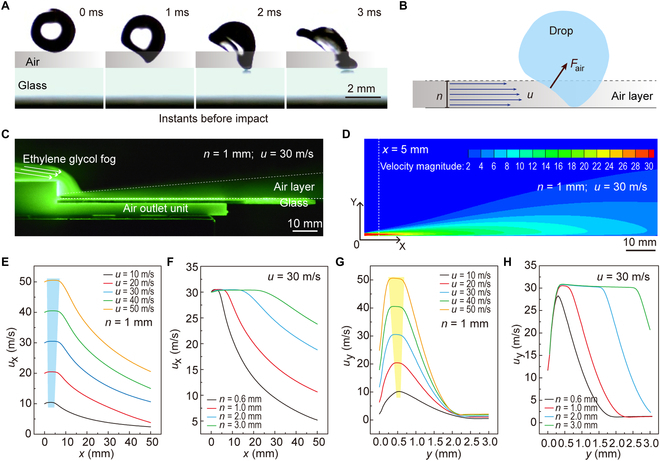
Mechanism of aerodynamic repellency of impacting drop. (A) Selected high-speed images showing the dynamics of instants before (*D* ~ 2.5 mm) impacting on a glass surface. (B) Schematic diagram of the force analysis of the impacting drop when it falls to the air layer. (C) The morphology of the air layer observed in an experiment. (D) Air layer morphology and velocity distribution obtained by numerical simulation. (E) Attenuation of the airflow velocity *u_x_* along the *x* direction with different initial airflow velocities at *n* = 1 mm. The blue area indicates the range where the *u_x_* can maintain stability. (F) Attenuation of the airflow velocity along the *x* direction with different thicknesses (*n*) of the air outlet at *u* = 30 m/s. (G) Attenuation of the airflow velocity along the *y* direction at the impacting point (*X* = 5 mm) with different initial airflow velocities at *n* = 1 mm. The yellow area denotes the range where the *u_y_* can remain stable. (H) Attenuation of the airflow velocity along the *y* direction at the impacting point (*X* = 5 mm) with different thicknesses (*n*) of the air outlet at *u* = 30 m/s.

To reveal the drop repulsion mechanism on the air layer surface, we focused on the interaction between the drop and air layer at the instant of impact. As shown in Fig. 4A, once the drop falls to the air layer, lateral deformation immediately occurs. Such asymmetric deformation arises from the strong dynamic pressure exerted by the air layer on the drop, causing the formation of a wedge-shaped air layer beneath the drop [35]. At the deformed inclined liquid–gas interface, a force *F*_air_ perpendicular to the interface is generated (Fig. 4B). The order of the aerodynamic force *F*_air_ equals the average dynamic pressure of the air in the gas layer (*ρ*_a_*u*^2^, denoting *ρ*_a_ as the density of air) multiplied by the surface area of the deformed portion of drop (*nR*), i.e.,

$$F_{\mathrm{air}}=\rho_{a}u^{2}\mathrm{nR}$$ (1)

The upward vertical component of the force *F*_air_ prevents the drop from falling further and lifts the liquid upward. Meanwhile, the horizontal component of the *F*_air_ participates in the movement of the drop along the direction of the airflow. Finally, the drop rebounds and leaves the entire surface under the action of the air layer.

The deformation of the drop induced by *F*_air_ is supposed to be responsible for the aerodynamic repellency and must occur fast enough to avoid contact. The deformation time *τ*_d_ can be obtained by balancing the aerodynamic force *F*_air_ with the viscous force

$$F_{\mathrm{vis}}=\eta dv/d\mathrm{yA}$$ (2)

where *η* is the viscosity coefficient of the water drop, d*v*/d*y* = R/(*nτ*_d_) is the gradient of velocity change, *A* = *mn* is the contact area. This yields

$$\tau_{d}=\eta m/\left(\rho_{a}{\mathrm{nu}}^{2}\right)$$ (3)

Only when the deformation time of the drop is shorter than the crashing time *τ*_c_ = *R*/*v*, drop rebounding is possible. For *τ*_c_ > *τ*_d_, we can obtain

$$f(u,\;n)=n^{0.5}u>(\eta m/(R\rho_{a}){)}^{0.5}v^{0.5}$$ (4)

On the other hand, the vertical component of the aerodynamic force *F*_air_ is required to be sufficient to overcome the inertia of the impacting drop. For the impacting drop to decelerate from *v* to 0 in 2*R*/*v* time, the magnitude of the inertial force for drop impact is *ρ*_w_*Rv*^2^. If the *F*_air_ is larger than the drop-impacting inertia force, the drop can bounce off the air layer surface, that is,

$$u>{\left(\rho_{w}D/\left(\rho_{a}n\right)\right)}^{0.5}v$$ (5)

i.e.,

$$f\left(u,n\right)=n^{0.5}u>{\left(\left(\rho_{w}D\right)/\rho_{a}\right)}^{0.5}v$$ (6)

which agrees with our experimental results shown in Fig. 3.

The thickness of the air layer *n* affects the velocity threshold u* for the drop to rebound (Fig. 3 and Fig. S3C). Equations 5 and 6 reveal that both the drop scale *D* and the air layer thickness *n* are in millimeters. Thus, the airflow velocity *u* required for the drop to rebound must be no more than approximately (*ρ*_w_/*ρ*_a_)^0.5^ ≈ 30 times the drop impact velocity *v*, which depends on the competition between the 2 forms of inertia: the drop inertia and the air inertia. This threshold is consistent with the phase diagram shown in Fig. S3C, where for the air layer thickness of *n* = 3 mm and 2 mm, when *u* < ~30 *v*, the direct wetting mode occurs, and when *u* > ~30 *v*, the bouncing or mixed mode occurs. However, for *n* = 1 mm and 0.6 mm, u* must be 55 to 70 times higher than the impact velocity *v* for the impact drop to rebound. This discrepancy is speculated to be due to the existence of the boundary layer and the attenuation of airflow velocity. Additionally, the boundary layer thickness and airflow velocity attenuation change with different initial airflow velocities, leading to u* being different even at the same air outlet and impacting location. Hence, the velocity threshold u* for drop rebound at different air layer thicknesses *n* differs.

To verify the above prediction, experimental observation and theoretical simulation are carried out for the morphology and velocity distribution of the air layer. To observe the air layer morphology, a green laser (532 nm, 30 MW) was placed in the center of the air outlet along the airflow direction, where the laser surface was perpendicular to the horizontal plane. When the air layer was introduced, a glycol smoke generator (1,000 W) was turned on allowing the glycol smoke to flow to the air outlet. Then, the airflow at the air outlet drives the glycol smoke to flow, showing clear air layer morphology, as shown in Fig. 4C and Fig. S4 (*u* = 30 m/s, *n* = 1 mm). As the distance increases, the airflow slowly diffuses upward and forward, and the air layer thickness changes.

To further explore the velocity distribution of the air layer, we conducted the numerical simulation of the air layer using the commercial fluid simulation software ANSYS Fluent (5.6 version), as detailed in Materials and Methods. The air layer morphology obtained by postprocess software is consistent with the experimental observation results (taking *u* = 30 m/s, *n* = 1 mm as an example, as shown in Fig. 4D). The simulation results accurately depict the velocity distribution in the air layer, which primarily maintains the airflow velocity in the middle of the air layer close to the air outlet and gradually decreases with increasing distance from the air outlet due to the existence of the boundary layer and diffusion into the surrounding air. To quantify the attenuation law of airflow velocity, a series of numerical simulations for the air layer with different *n* and *u* adopted in the above experiments were conducted. The results show that the airflow velocity decreases with the increase of the distance from the air outlet and diffuses into the air for all conditions, as shown in Fig. S5. For the same *u*, the larger the air outlet thickness *n*, the slower the attenuation of the airflow velocity. Similarly, for the same *n*, the larger the initial airflow velocity *u*, the slower attenuation of the velocity.

To quantitatively describe the rules, we extracted the air velocity *u*_x_ along *x* directions on the center line of the air outlet (*y* = *n*/2) and obtained the attenuation curve of air layer velocity under different air outlet thicknesses and air velocity, as shown in Fig. 4E and F and Fig. S6A. To explore the actual thickness n* of the air layer, taking the impact position *x* = 5 mm in the experiments as an example, the change curves of the airflow velocity along the *y* direction *u*_y_ at *x* = 5 mm were described in Fig. 4G and H and Fig. S6B. Here, the range of *y* when *u*_y_ is greater than 80% of *u* can be taken as the effective thickness n* of the air layer. Figure S6C shows the value of n* achieved with the change of *u*. The result demonstrates that smaller *n* is unfavorable to the maintenance and stability of airflow velocity. For example, when *n* is only 0.6 mm, n* is only 0.1 mm (*u* = 10 m/s), which is almost zero. This means that almost all energy provided by the air layer is lost. In contrast, when *n* is larger, the airflow velocity keeps relatively stable. For example, when *n* is 3 mm, n* reaches 2.75 mm (*u* = 50 m/s), resulting in a small energy loss. Therefore, both the n* and *u* decide the stability or attenuation of the airflow velocity. In general, when considering the airflow flow threshold provided, the larger *n* and *u* result in slower velocity attenuation, smaller energy loss, and easier repulsion of drops by the air layer surface. These results help to understand the maintenance of airflow velocity under various experimental parameters.

### Drop impacting on inclined air layer substrate

**Fig. 5.**
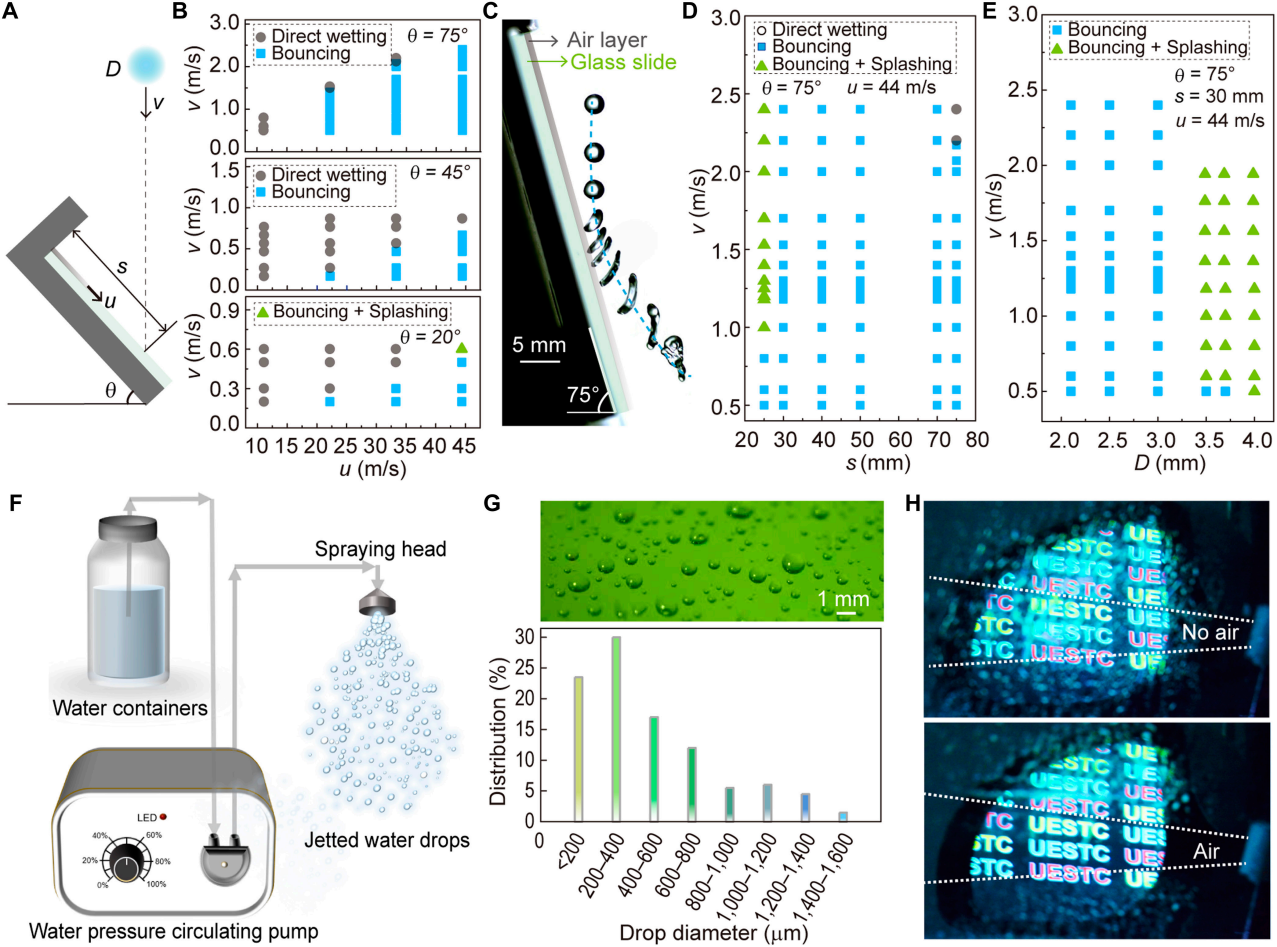
Aerodynamic repellency of impacting drop in rainproof side windshield. (A) Side-view schematic diagram of a drop impacting on an inclined air layer surface. (B) The effect of the surface inclination angle (*θ*) on impacting outcomes (*n* = 1 mm). (C) The time-lapse trajectory of a drop impacting and rebounding off on an inclined air layer surface with *θ* = 75°, *s* = 40 mm, *u* = 33 m/s, and *v* = 1 m/s. (D) The effect of impacting position (s) on impacting results at *θ* = 75°, *u* = 44 m/s. (E) The effect of the drop diameter (*D*) on impacting outcomes at *θ* = 75°, *u* = 44 m/s, and *s* = 30 mm. Laboratory simulation of driving in rainy scenes: (F) Schematic diagram of raindrop generator device. (G) Raindrop size distribution. (H) When the air layer was introduced to the surface of the side windshield, the raindrops were repelled achieving a clear vision.

Considering various situations in practical application, we studied the effect of air-layer surface incline angle, the action range of the air layer, and the drop size on impacting results. Figure 5A shows a schematic diagram of the impact of liquid drops on an inclined air layer surface, where *θ* is the inclination angle between the air layer surface and the horizontal plane, *s* is the distance between the impact position of liquid drops and the air outlet, denoting *s* as the action length of the air layer, and the thickness of the air outlet is fixed as *n* = 1 mm. Figure 5B shows the impact results at different inclination angles, where the nonwetting area (blue) of the glass surface means that the drops are repulsed and rebound after impacting the air layer surface. The results show that the ease of drop removal increased with an increase in the inclination angle. This is because when the drop impacts with *v*, the component of *v* perpendicular to the inclined surface, *v*cos*θ*, decreases with increasing *θ* value. Therefore, a larger surface inclination angle benefits in improving the threshold of a drop impacting velocity for removal. Meanwhile, once the impact drops rebound, the component of *v* downward along the inclined surface (*v*sin*θ*) participates and helps the rebound drops move along the surface under the aerodynamic force of the air layer until the drops are completely off the surface, achieving the long-term nonwetting state of the air layer surface. Taking *θ* = 75°, *v* = 1 m/s, and *u* = 33 m/s as an example, when the drop with a diameter of 2.5 mm impacts 40 mm away from the air outlet, the drop can completely leave the entire glass surface after rebound under the action of the air layer (Fig. 5C and Movie S5).

To further verify the influence of the action length of the air layer, we investigated the impact results under different impacting positions *s* = 20, 30, 40, 50, 70, and 75 mm (*θ* = 75°, *D* = 2.5 mm, *n* = 1 mm, and *u* = 44 m/s), as shown in Fig. 5D. The results demonstrate that despite the attenuation of airflow velocity, the air layer glass surface can achieve a wide range of rebounding after drop impact, ensuring the nonwetting of the entire surface. Figure S7 shows the time-lapse bouncing dynamics of an impacting drop at *s* = 75 mm. Finally, the influence of drop size on impacting results was explored. Impinging drops with diameters of *D* = 2.1 to 4.0 mm were obtained by using needles of different specifications. Figure 5E and Fig. S8 show the diagram of drops with different diameters impacting the air layer surface at *u* = 44 m/s, *θ* = 75°, *s* = 30 mm, and *n* = 1 mm. The results show that the smaller the drop size, the easier the drop to be removed from the surface under the action of the air layer. This is because a smaller drop size provides lower impacting energy, thereby reducing the threshold of the energy required by the air layer. Generally, larger air outlet thickness *n* and air velocity *u* can maintain the stability of airflow velocity, and a larger surface inclination angle *θ* and a smaller drop size can improve the threshold of the drop impacting velocity for removal. The air layer surface can achieve a wide range of rebounding after drop impact and ensure the nonwetting of the entire surface, even with the attenuation of airflow velocity. These findings can provide a valuable reference for the design and application of antiwetting technology in various practical scenarios.

### Application of the air layer surface in automobile side windshield

Based on the excellent repellency performance to impacting drops, air layer surface may provide a new solution and feasible method for resolving the raindrop adhesion on automobile side windshields. Here, an in-lab rainy-day simulation system was set up to verify the rainproof effect of the air layer applied to the side windshield. The system mainly includes a rain generator, an air layer introduction device, an automobile rearview mirror, and a large glass (70 cm × 50 cm × 1 cm) that acts as an automobile side windshield. The raindrop generator was realized by a micropressure peristaltic pump combined with a drop nozzle (Fig. 5F). To investigate the size range of the raindrop produced by the generator, a high-speed camera was used to record the drop-falling process after they were sprayed from the nozzle. Figure 5G shows the falling raindrops attached to a glass surface and the drop size distribution ranging from ~150 μm to ~2.5 mm, which falls within the range of light to moderate rain.

To simulate the scenario more realistically, the rearview mirror and side window were kept at normal driving positions. A camera (D7100, Nikon) was used to record the driver’s view field when observing the scene in a rearview mirror through the side windshield. A projector (CB-X03, Epson) was used to set the projection subtitles “UESTC” with common light colors such as red, yellow, blue, and green in the rear field of vision to simulate the effect of the light source. As shown in Fig. 5H, without introducing an air layer, the surface of the side windshield was wetted by the adhered raindrops, resulting in blurred color subtitles in the field of view of the rearview mirror. However, when an air layer is introduced onto the side windshield, the area covered by the air layer keeps clear. Since a certain distance is needed to install an air lead-in device on the car side windshield in practical application, the action length of the air layer should be explored, as shown in Fig. S9A. For the area where the driver needs to observe the rearview mirror through the side window (Fig. S9B), the maximum length is 10 to 15 mm (depending on the vehicle type). When the size of the air outlet is 5 mm × 1 mm, the air layer can reach the action length of ~30 cm under a certain airflow velocity (Fig. S9C). This demonstrates the potential and feasibility of this method for realizing rainproof performance on automobile side windshields.

## Conclusion

In summary, the repulsion and directional removal of impacting drops were realized by simply imposing an exogenous air layer on a solid substrate. By controlling the thickness and flow velocity of the air layer, in principle, the solid surface can be kept nonwetting under the continuous impact of a water drop or other liquids. This kind of rebound is based on the effect of dynamic pressure provided by the air layer. The vertical component of the aerodynamic force pushes the drop upward to rebound for one thing, but also, the horizontal component along the flow direction involves the long-distance movement of the drop on the surface. The dynamics of the vent can spend as a form of an aerodynamic Leidenfrost effect by controlling the air layer between the liquid drop and the solid surface. The effect is more productive for smaller drop sizes and more sloping surfaces. We finally applied this method to the surface of the car side windshield in the lab, showing a strong potential to solve the problem of driving safety caused by raindrop adhesion on the side windshield on rainy days. Such aerodynamic repellency of liquid drops has universal applicability, regardless of the physical properties of the solid surface and impacting drops.

## Materials and Methods

### Surface treatment and air layer introduction device printing

The glass slides (borosilicate glass material, 75 mm × 26 mm × 1 mm, MARIENFELD) were rinsed with ethanol (AR, ≥99.7%, HUSHI) and ultrapure water (18.2 MΩ/cm, from Synergy Water Purification System, Millipore SAS), respectively. The final glass surface exhibited a hydrophilic wetting state. The air layer introduction device was obtained by 3D printing technique (3D printer, 100-μm printing precision, i3 mega, ANYCUBIC) using polylactic acid as the printing material. The processing temperature was set as 200 °C.

### Drop-impacting experiments on air layer surface

The experimental device was set up as shown in Fig. 2B. The impinging drop was water, and the substrate was a glass slide with a thickness of 1 mm. The upper surface of the glass slide was flat with the lower part of the air outlet to avoid blocking the airflow. The drop-impacting dynamics were recorded by a high-speed camera (Photron, FASTCAM SA5 32G, Japan) with a frame rate of 10,000 fps. Before the drop-impacting experiment, the air compressor was opened for a while to ensure that the airflow through the glass surface was stable. The airflow velocity *u* is calculated by *u* = *Q*/*mn*.

### Numerical simulation of the air layer

We conducted the numerical simulation of the air layer using the commercial fluid simulation software ANSYS Fluent (5.6 version). The brief simulation steps are as follows: (a) Two-dimensional square grids are obtained using Gambit software, in which the grid computing domain is 100 mm × 100 mm. The size of the internal single grid is 5 to 20 μm, which is heterogeneous. The grid size near the wall and the air outlet is smaller. The boundary conditions are set as air inlet, wall, and pressure outlet. (b) During simulation, the max-equation model and RNG k-epsilon (2-equation) are adopted, and the calculation is performed by the Simplec method. To improve the calculation accuracy, adaptive densification of near-base surface mesh is carried out during modeling. The corresponding time step is set as 2.0 × 10^−6^ s. The airflow density and viscosity are set as 1.225 kg/m^3^ and 1.85 × 10^−5^ kg/(m·s), respectively. (c) To visualize the simulation results, computational fluid dynamics postprocessing software (Tecplot) is used to obtain the air layer morphology and velocity distribution.

## Data Availability

The data used to support the findings of this study are present in the paper and supporting materials. Additional data related to this paper may be requested from the authors.

## Supplementary Materials

Supplementary 1 Fig. S1. The poor mechanical stability of the micro/nanostructured superhydrophobic coating. Fig. S2. Air layer introduction device. Fig. S3. The impacting outcomes. Fig. S4. Experimental observation of the air layer. Fig. S5. Numerical simulation of the air layer at different *u* and *n*. Fig. S6. Simulation results analysis of the air layer at different *u* and *n*. Figs. S7 to S8. Drop-impacting dynamics on an inclined air layer surface. Fig. S9. The action range of the air layer surface is required when applied to the automobile side windshield.

Supplementary 2 Movie S1. Drop impacting on an air layer surface: Bouncing mode. Movie S2. Drop wetting on a smooth surface without an air layer. Movie S3. Drop impacting on an air layer surface: Direct wetting mode. Movie S4. Drop impacting on an air layer surface: Bouncing and splashing mode. Movie S5. Drop bouncing on an inclined air layer surface.
