# Supplementary material for: Aerodynamic Super-Repellent Surfaces
Source: Research (Wash D C). 2023 Apr 19;2023:0111. doi: 10.34133/research.0111 (PMC10202376; doi:10.34133/research.0111)
Supplement: Supplementary 1 — Fig. S1. The poor mechanical stability of the micro/nanostructured superhydrophobic coating. Fig. S2. Air layer introduction device. Fig. S3. The impacting outcomes. Fig. S4. Experimental observation of the air layer. Fig. S5. Numerical simulation of the air layer at different u and n. Fig. S6. Simulation results analysis of the air layer at different u and n. Figs. S7 to S8. Drop-impacting dynamics on an inclined air layer surface. Fig. S9. The action range of the air layer surface is required when applied to the automobile side windshield. [file research.0111.f1.docx]

**Supporting materials**


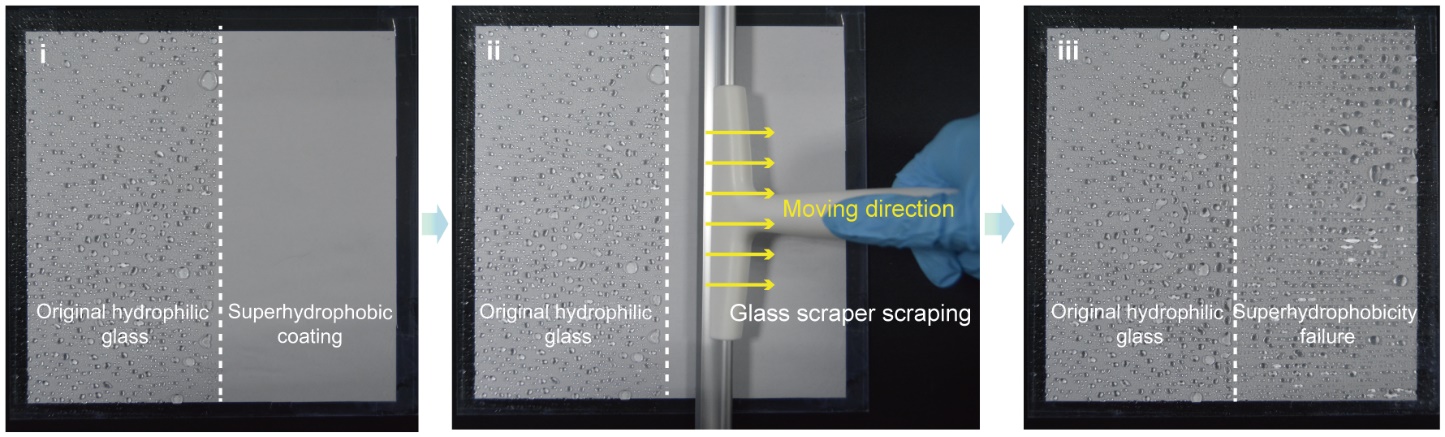


**Figure S1**. **The poor mechanical stability of the micro-/nano- structured superhydrophobic coating**. (i) A glass slide, half of which remains hydrophilic and half was sprayed with a commercial superhydrophobic coating staying dry after being sprayed with water. (ii) A glass scraper scraped the superhydrophobic area one time. (iii) The superhydrophobic area became hydrophilic.


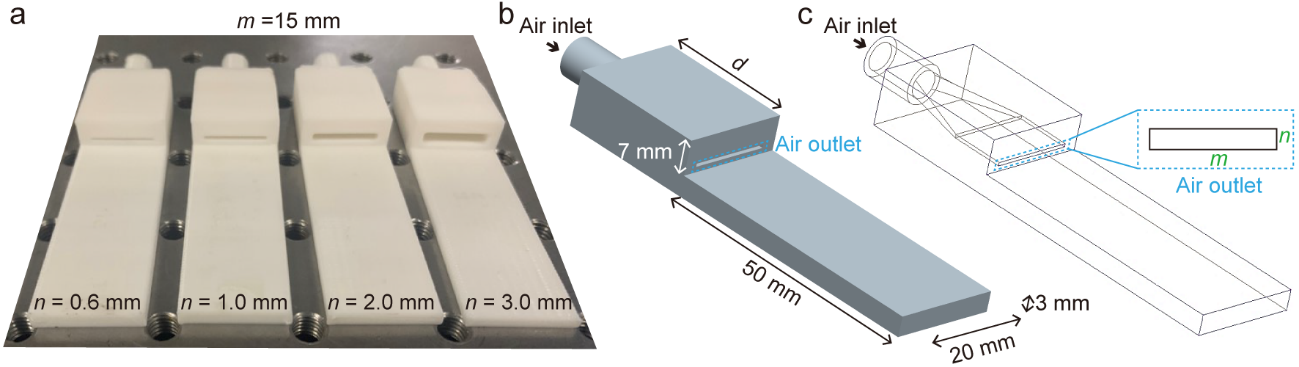


**Figure S2**. **Air layer introduction device.** (a) 3D printing device with different parameters. (b) Schematic diagram of the air-layer introduction device including air inlet, air outlet and substrate support. (c) Internal structure connection between the air inlet and outlet.


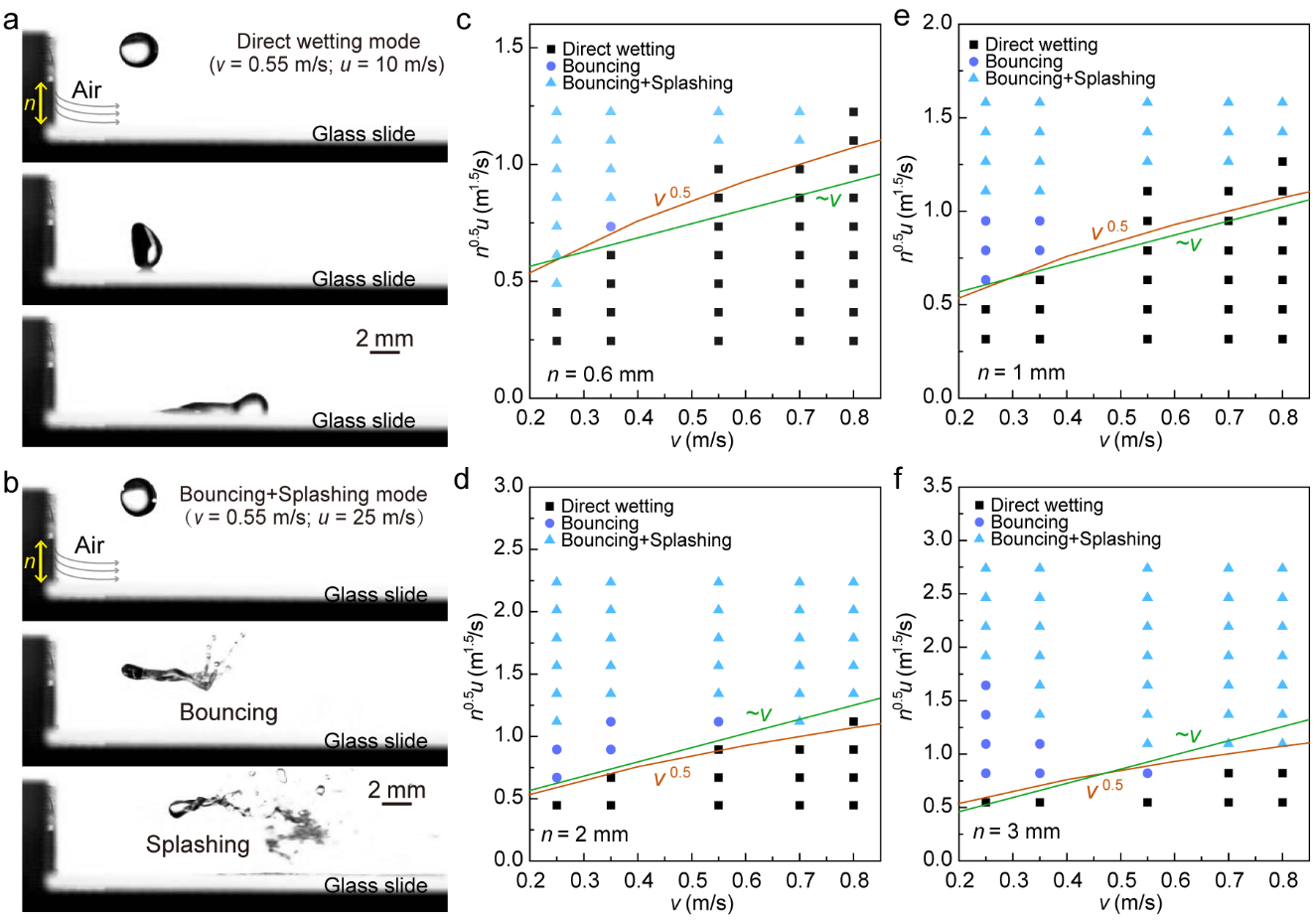


**Figure S3**. **The impacting outcomes.** (a) When the airflow velocity u is too small, the drop presents a direct wetting mode (*v* = 0.55 m/s; *u* = 10 m/s, *n* = 3 mm). (b) When *u* is too high, the drop splashes after the impact and rebound (*v* = 0.55 m/s; *u* = 25 m/s, *n* = 3 mm), showing a mixed mode including bouncing and splashing. (c-f) The effect of *u*, drop impacting velocity *v* and the thickness of air outlet *n* on impacting outcomes, shows that larger *n* benefits the rebound of the impacting drop.


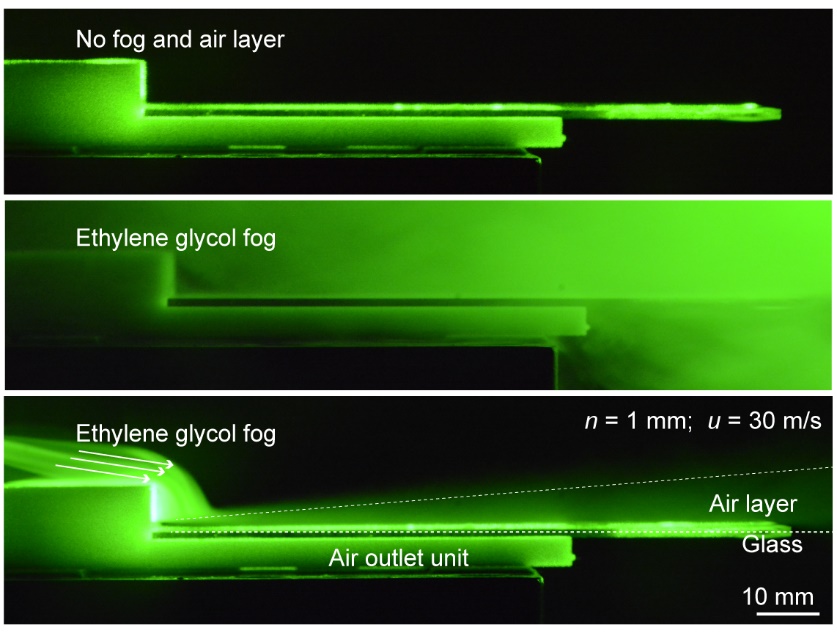


**Figure S4**. **Experimental observation of the air layer.** Air-layer morphology at *u* = 30 m/s, *n* = 1 mm.


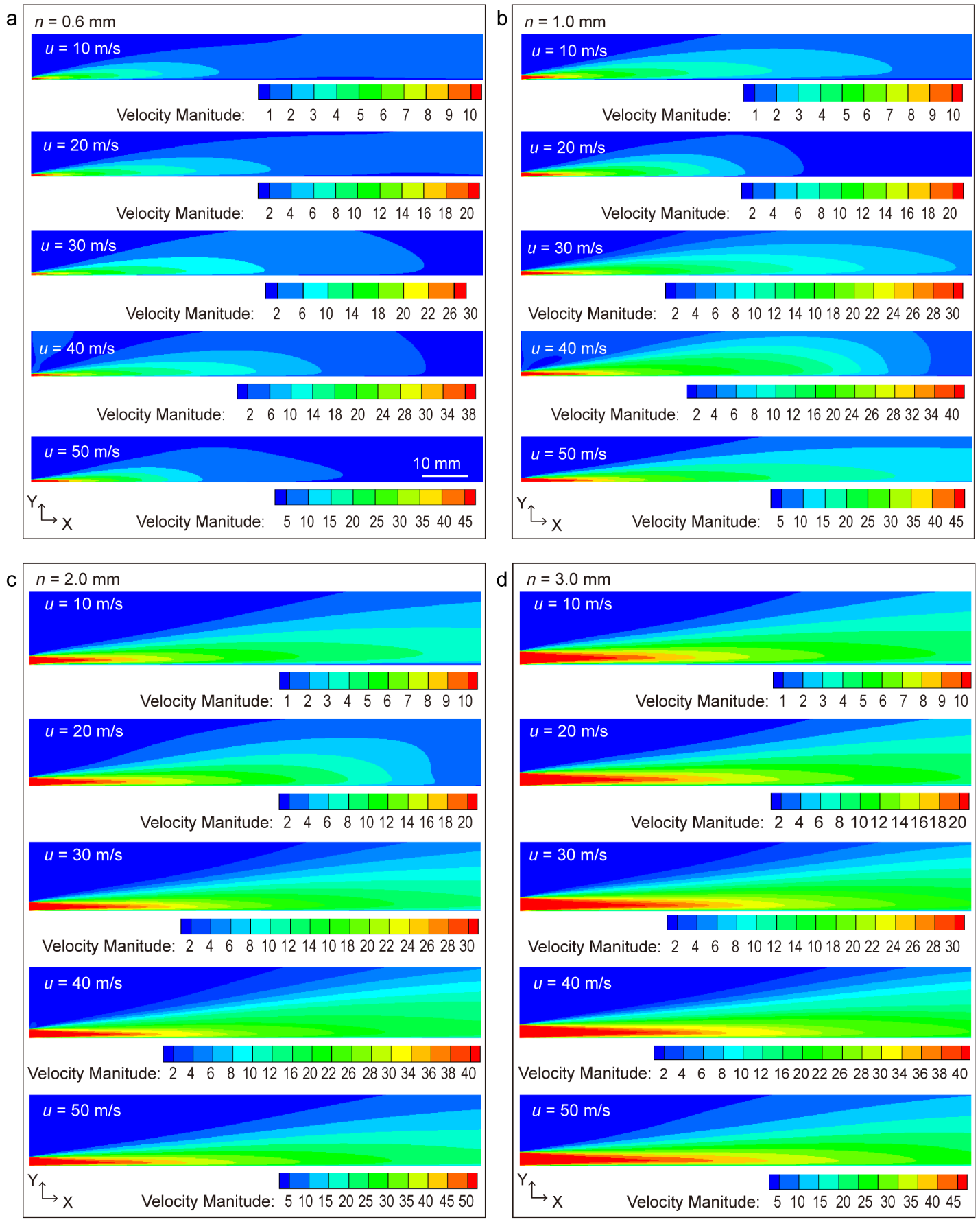


**Figure S5**. **Numerical simulation of the air layer at a different initial velocity of the air layer *u* and air outlet thickness *n*.** The distribution of the airflow velocity and air-layer morphology at u ranged from 10 m/s to 50 m/s: (a) *n* = 0.6 mm, (b) *n* = 1.0 mm, (c) *n* = 2.0 mm, (d) *n* = 3.0 mm.


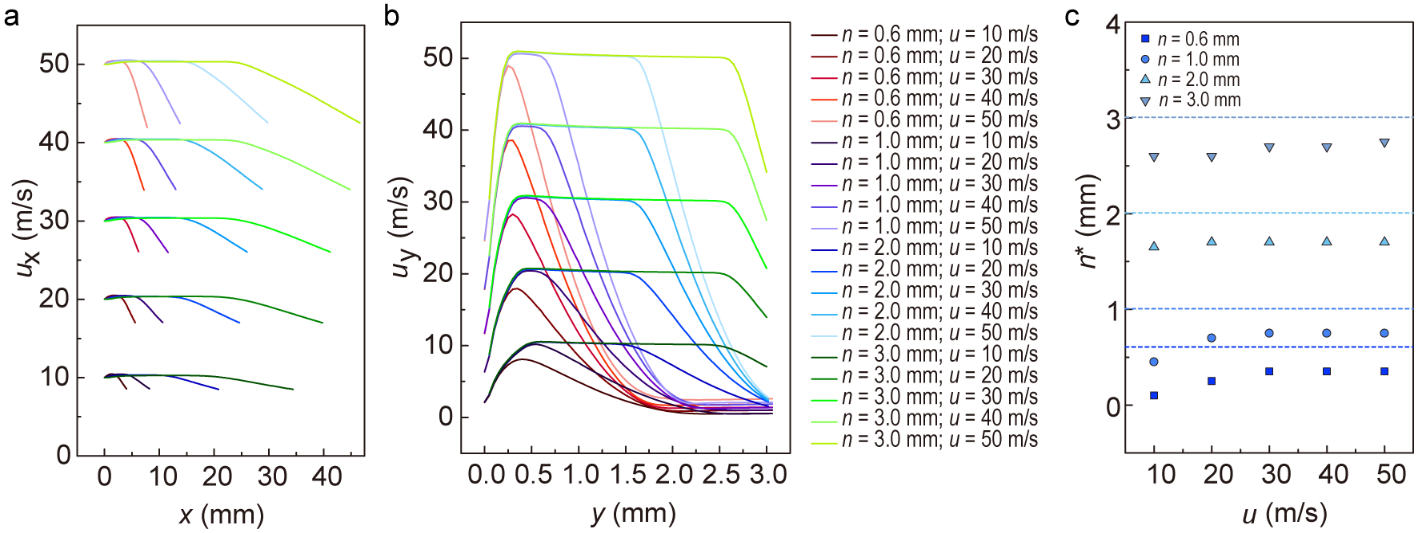


**Figure S6**. **Simulation results of the air layer at different *u* and *n*.** (a) Distribution of the airflow velocity along the *x*-direction. (b) Distribution of the airflow velocity along the *y*-direction. (c) The effective air layer thickness *n** at different airflow velocities equals 80% initial velocity.


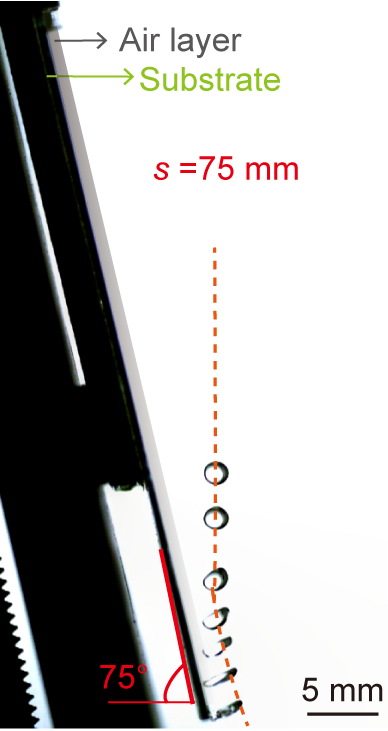


**Figure S7.** **Drop impacting dynamics on an inclined air-layer surface.** The time-lapse bouncing dynamics of an impacting drop at *s* = 75 mm (*n* = 1 mm; *v* = 2.35 m/s; *u* = 44 m/s).


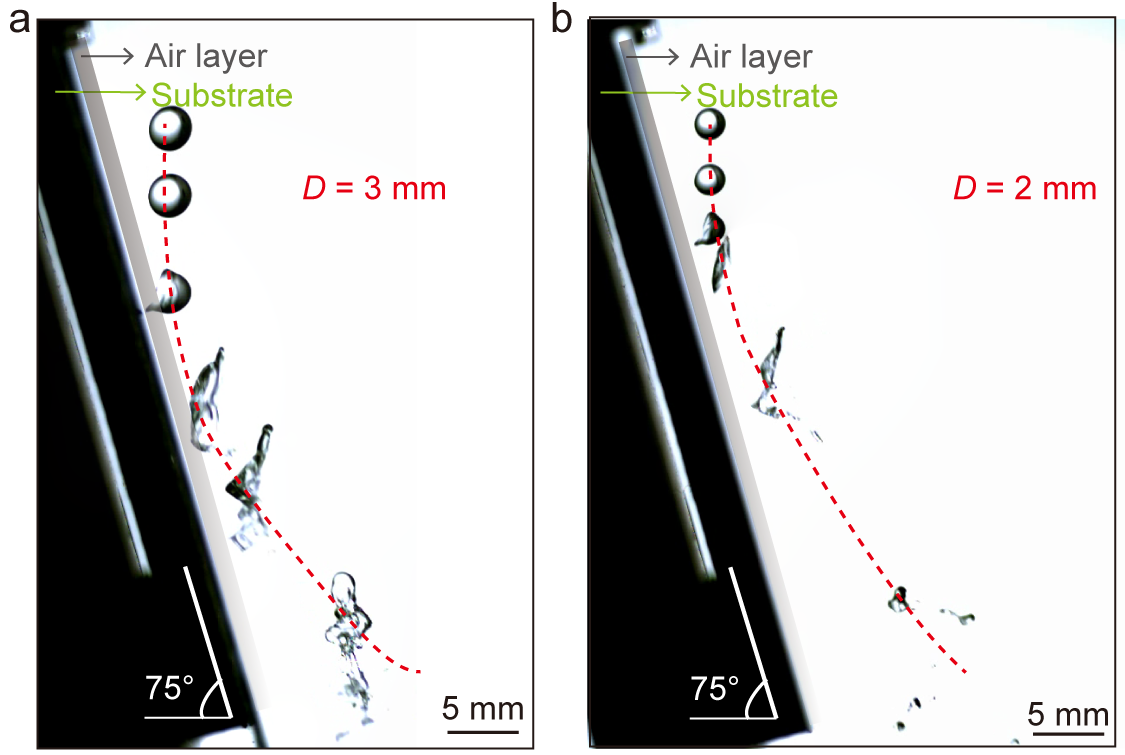


**Figure S8.** **Drop impacting dynamics on an inclined air-layer surface.** The time-lapse bouncing dynamics of an impacting drop at *D =* 3 mm and 2 mm (*s* = 40 mm; *n* = 1 mm; *v* = 2.35 m/s; *u* = 44 m/s), respectively.


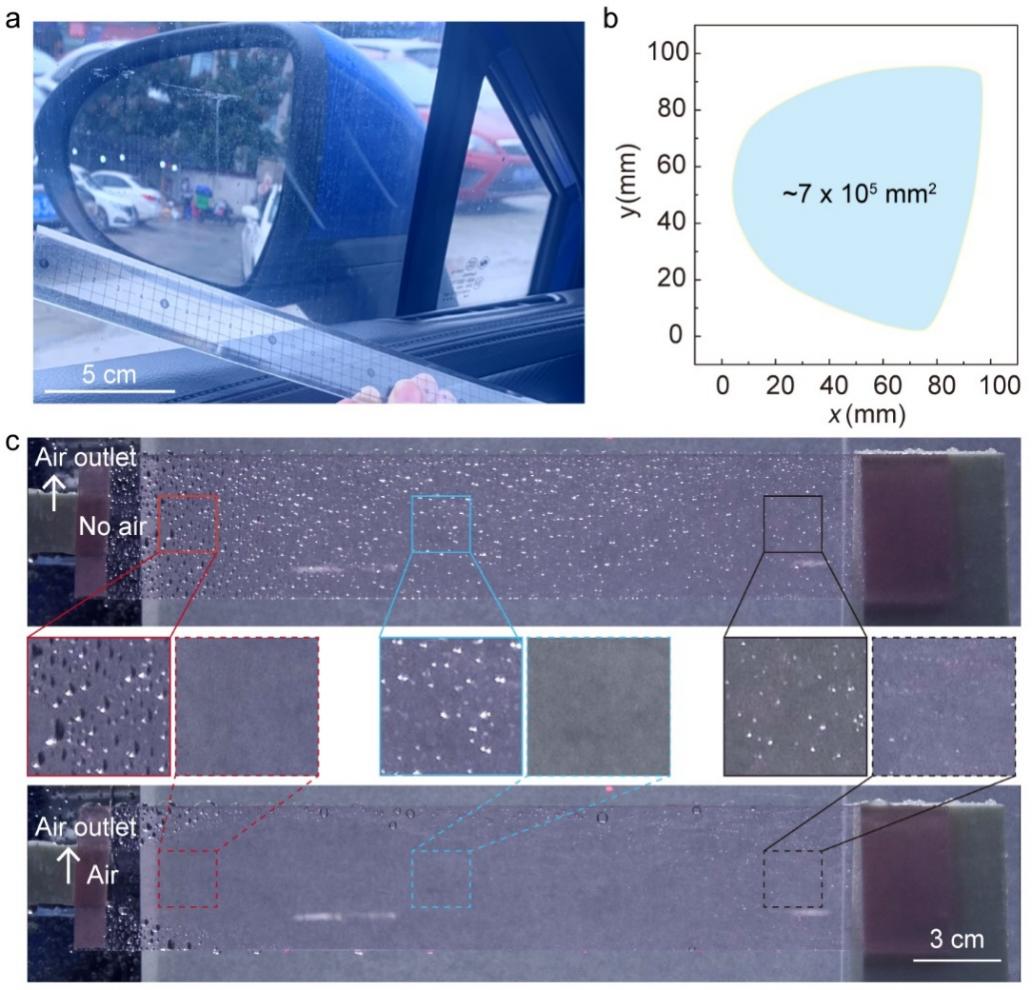


**Figure S9**. **The action range of the air-layer surface is required when applied to the automobile side windshield.** (a-b) Measurement of the clear range of the side windshield required for an unobstructed view of the rearview mirror from the driver's perspective. (c) The action length of the air layer can reach 30 cm at *n* = 1 mm, *u* = 80 m/s. This proves the feasibility of applying the air layer to the surface of the automobile side windshield to prevent the adhesion of raindrops.
