# Supplementary figures and images for: Aerodynamic Super-Repellent Surfaces
Source: Research (Wash D C). 2023 Apr 19;2023:0111. doi: 10.34133/research.0111 (PMC10202376; doi:10.34133/research.0111)

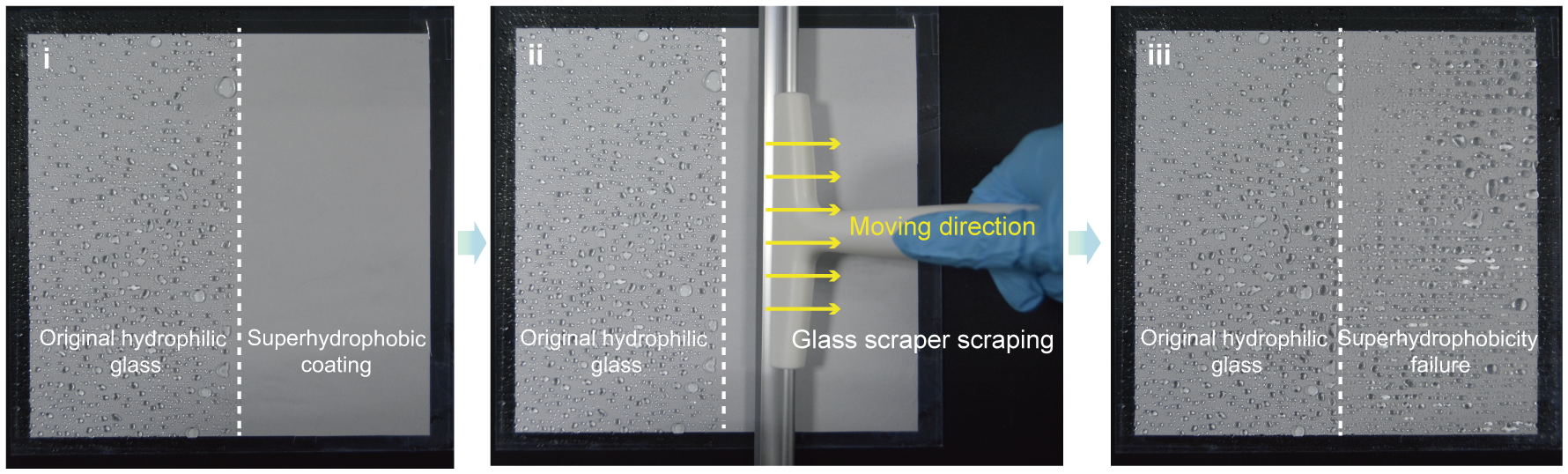

Supplement: Supplementary 2 — Movie S1. Drop impacting on an air layer surface: Bouncing mode. Movie S2. Drop wetting on a smooth surface without an air layer. Movie S3. Drop impacting on an air layer surface: Direct wetting mode. Movie S4. Drop impacting on an air layer surface: Bouncing and splashing mode. Movie S5. Drop bouncing on an inclined air layer surface. [file research.0111.f2.zip › Figure S1.tif]

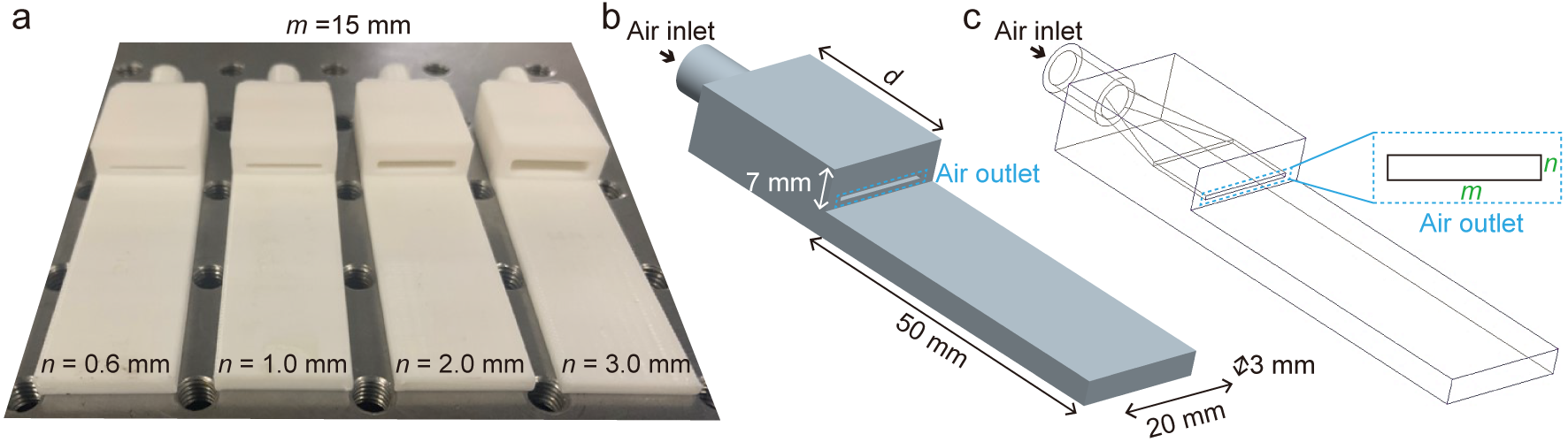

Supplement: Supplementary 2 — Movie S1. Drop impacting on an air layer surface: Bouncing mode. Movie S2. Drop wetting on a smooth surface without an air layer. Movie S3. Drop impacting on an air layer surface: Direct wetting mode. Movie S4. Drop impacting on an air layer surface: Bouncing and splashing mode. Movie S5. Drop bouncing on an inclined air layer surface. [file research.0111.f2.zip › Figure S2.tif]

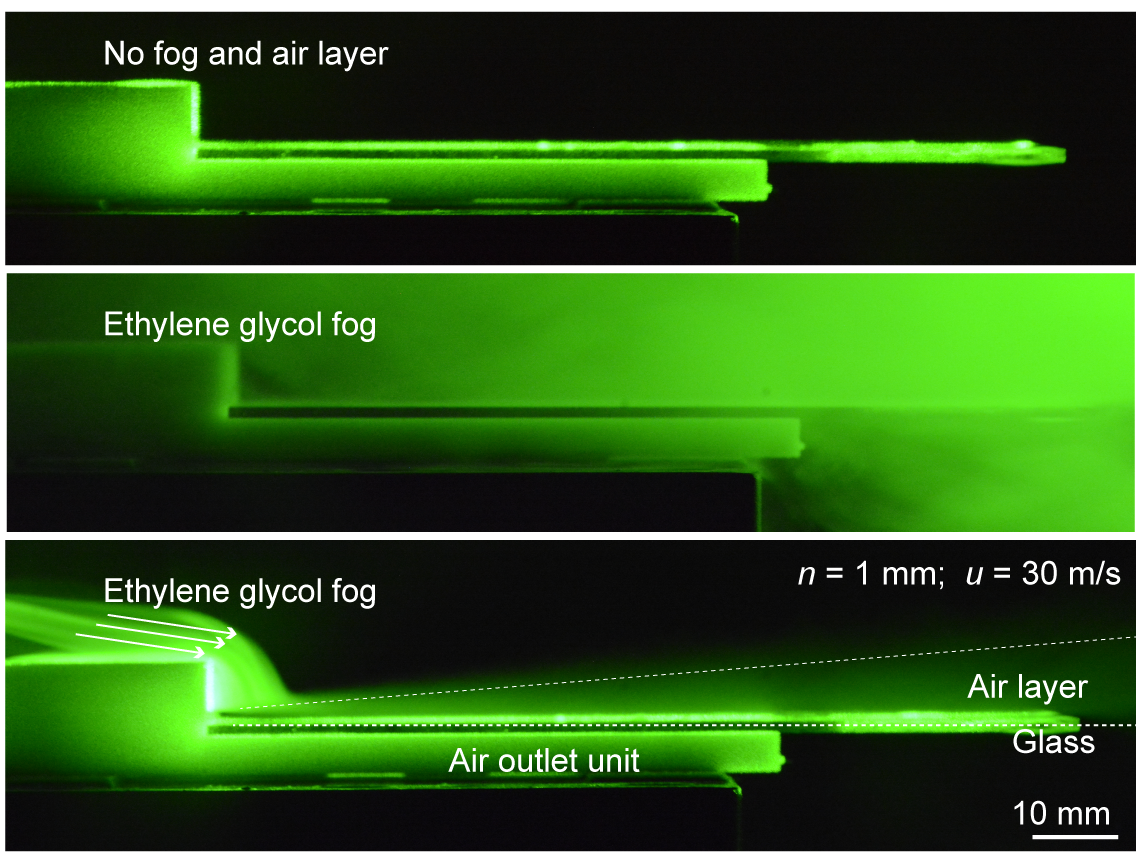

Supplement: Supplementary 2 — Movie S1. Drop impacting on an air layer surface: Bouncing mode. Movie S2. Drop wetting on a smooth surface without an air layer. Movie S3. Drop impacting on an air layer surface: Direct wetting mode. Movie S4. Drop impacting on an air layer surface: Bouncing and splashing mode. Movie S5. Drop bouncing on an inclined air layer surface. [file research.0111.f2.zip › Figure S4.tif]

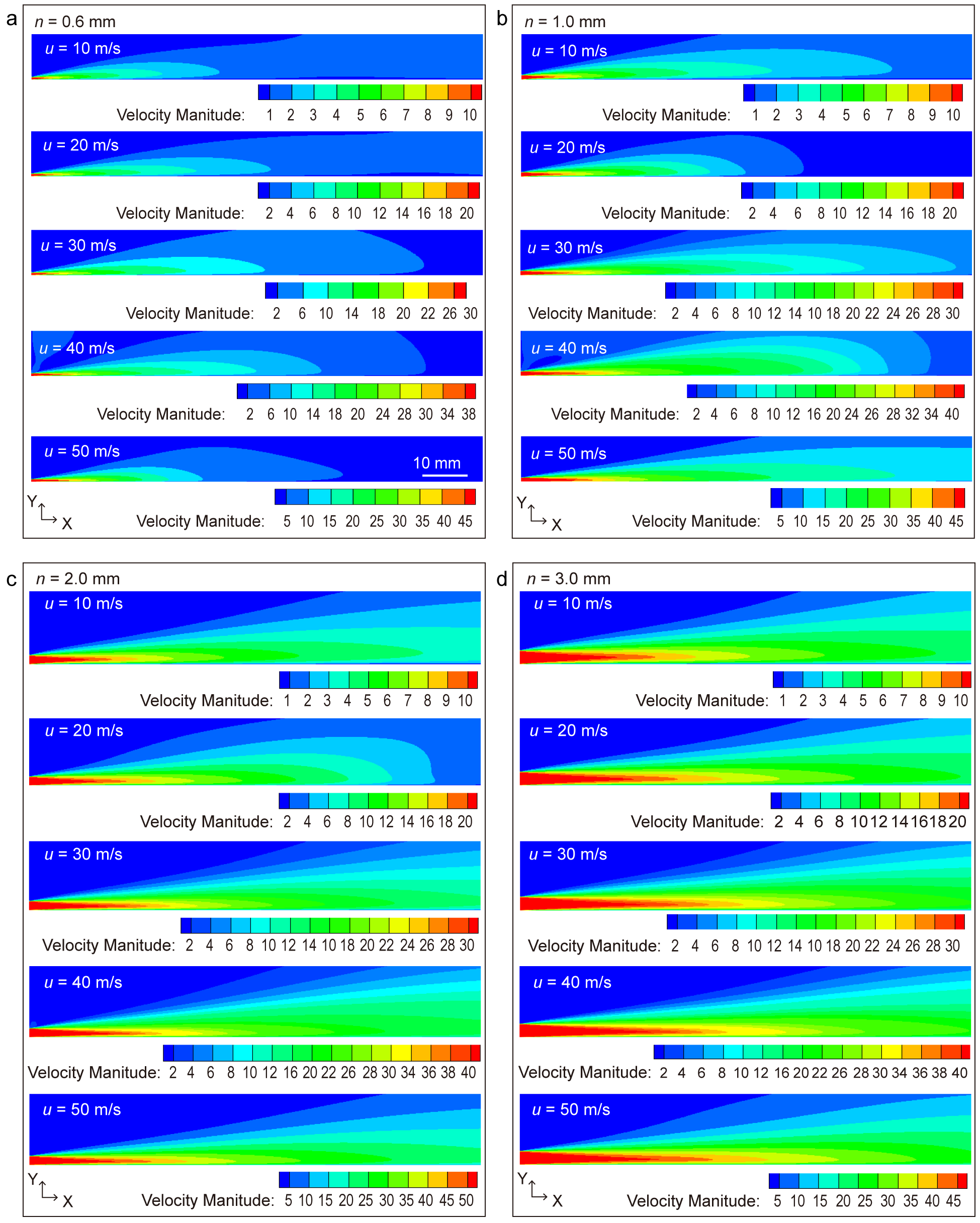

Supplement: Supplementary 2 — Movie S1. Drop impacting on an air layer surface: Bouncing mode. Movie S2. Drop wetting on a smooth surface without an air layer. Movie S3. Drop impacting on an air layer surface: Direct wetting mode. Movie S4. Drop impacting on an air layer surface: Bouncing and splashing mode. Movie S5. Drop bouncing on an inclined air layer surface. [file research.0111.f2.zip › Figure S5.tif]

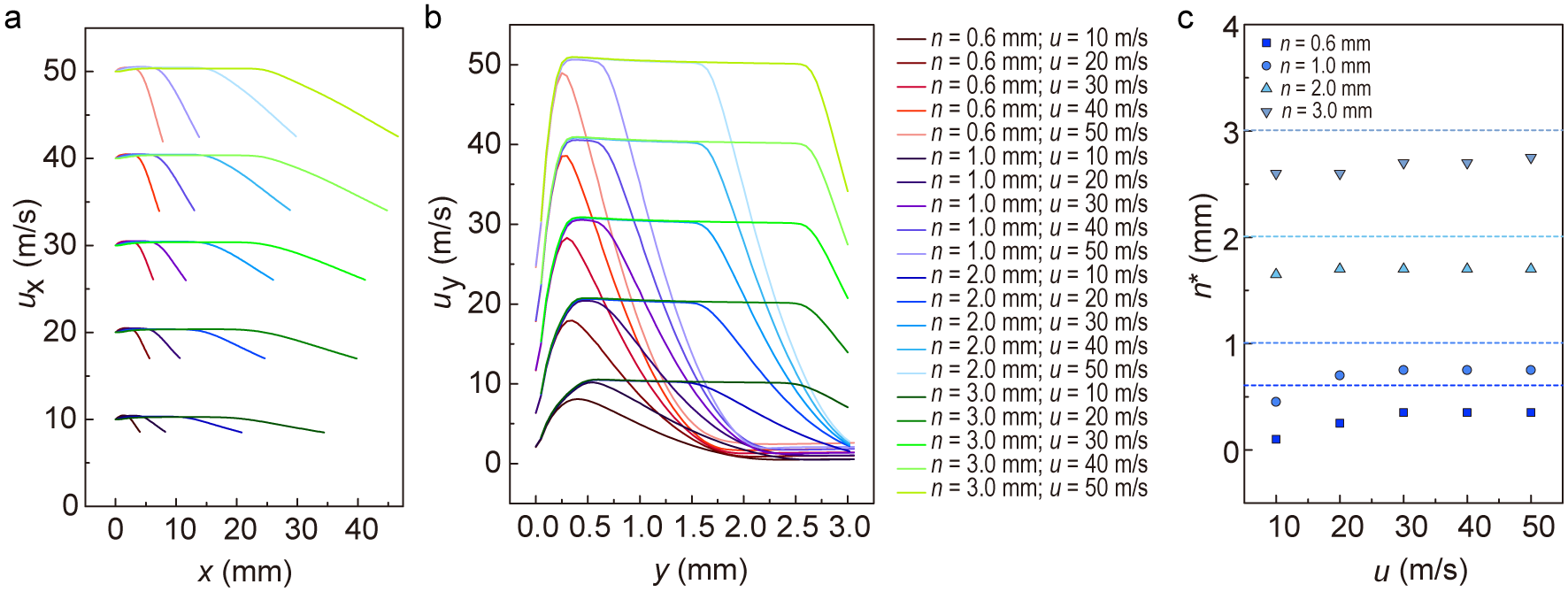

Supplement: Supplementary 2 — Movie S1. Drop impacting on an air layer surface: Bouncing mode. Movie S2. Drop wetting on a smooth surface without an air layer. Movie S3. Drop impacting on an air layer surface: Direct wetting mode. Movie S4. Drop impacting on an air layer surface: Bouncing and splashing mode. Movie S5. Drop bouncing on an inclined air layer surface. [file research.0111.f2.zip › Figure S6.tif]

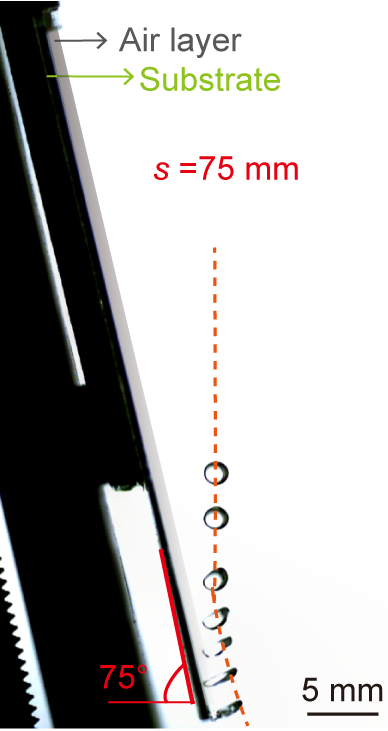

Supplement: Supplementary 2 — Movie S1. Drop impacting on an air layer surface: Bouncing mode. Movie S2. Drop wetting on a smooth surface without an air layer. Movie S3. Drop impacting on an air layer surface: Direct wetting mode. Movie S4. Drop impacting on an air layer surface: Bouncing and splashing mode. Movie S5. Drop bouncing on an inclined air layer surface. [file research.0111.f2.zip › Figure S7.tif]

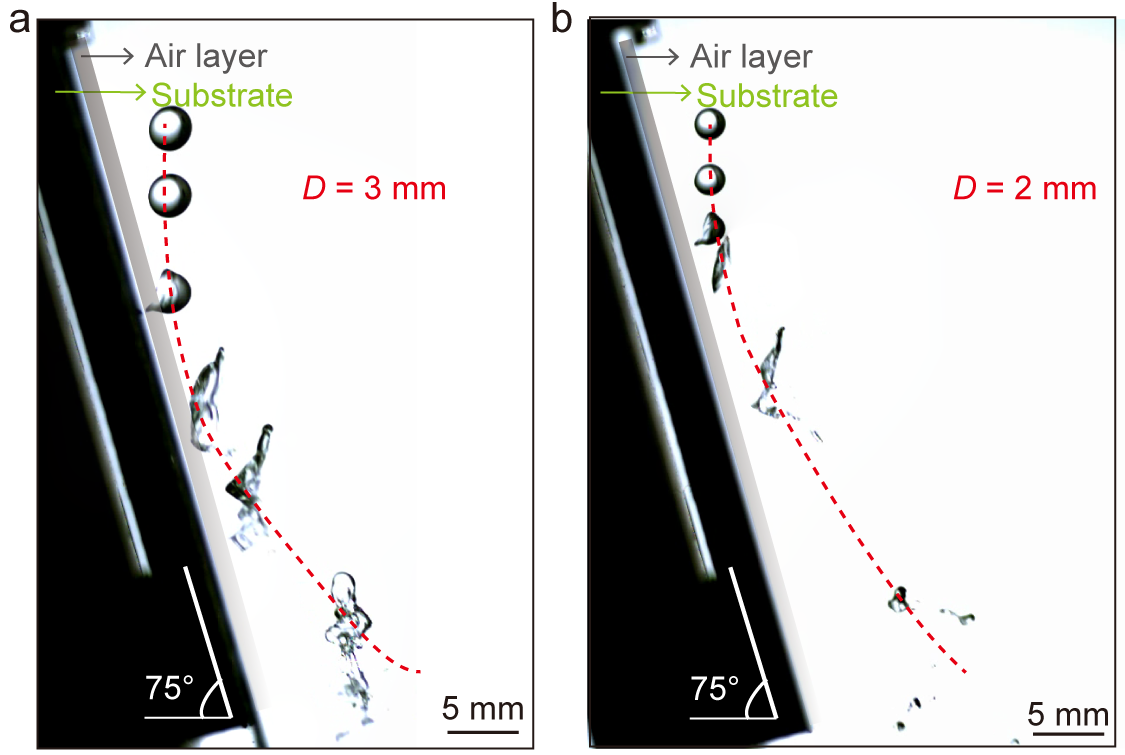

Supplement: Supplementary 2 — Movie S1. Drop impacting on an air layer surface: Bouncing mode. Movie S2. Drop wetting on a smooth surface without an air layer. Movie S3. Drop impacting on an air layer surface: Direct wetting mode. Movie S4. Drop impacting on an air layer surface: Bouncing and splashing mode. Movie S5. Drop bouncing on an inclined air layer surface. [file research.0111.f2.zip › Figure S8.tif]

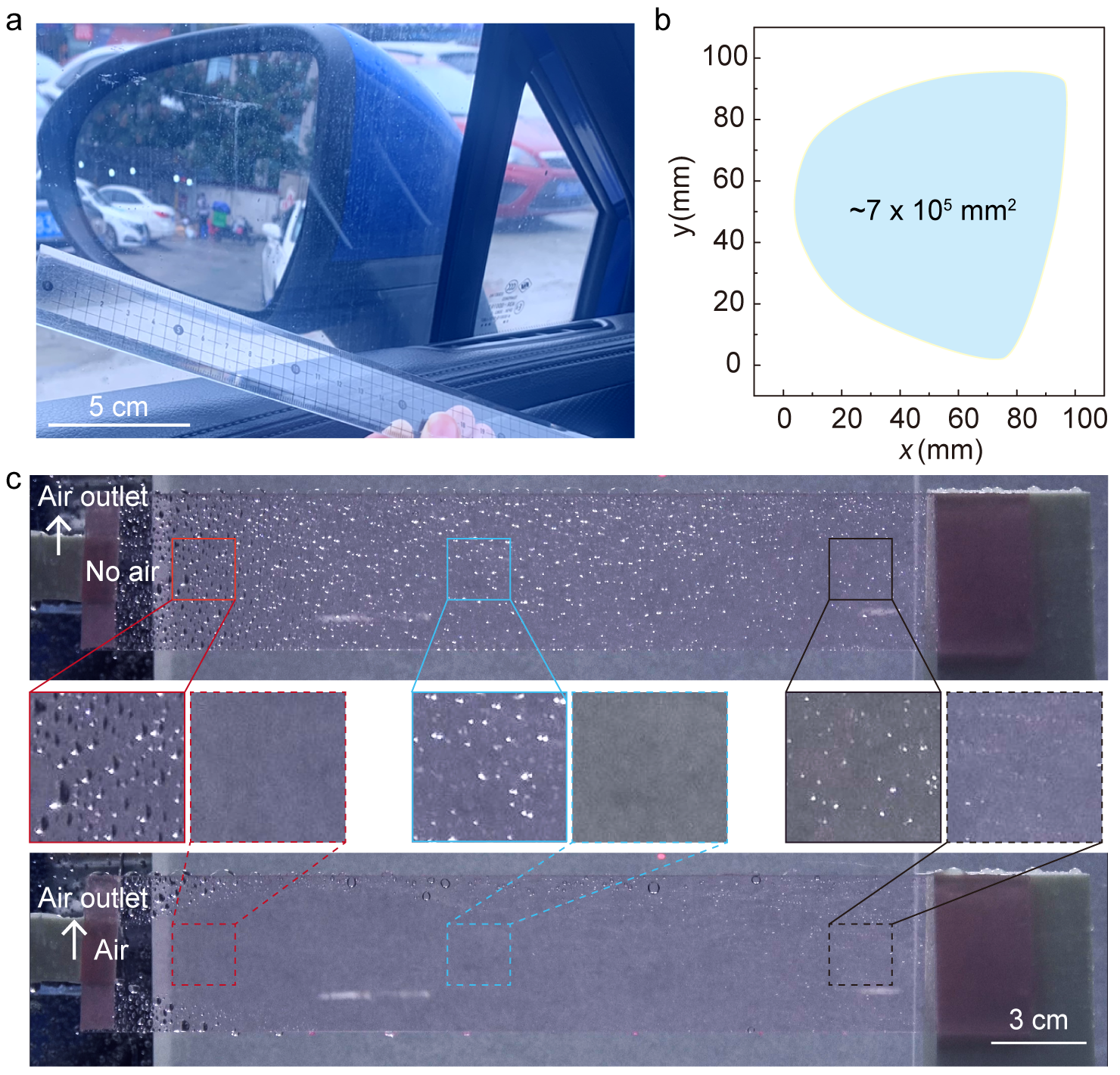

Supplement: Supplementary 2 — Movie S1. Drop impacting on an air layer surface: Bouncing mode. Movie S2. Drop wetting on a smooth surface without an air layer. Movie S3. Drop impacting on an air layer surface: Direct wetting mode. Movie S4. Drop impacting on an air layer surface: Bouncing and splashing mode. Movie S5. Drop bouncing on an inclined air layer surface. [file research.0111.f2.zip › Figure S9.tif]

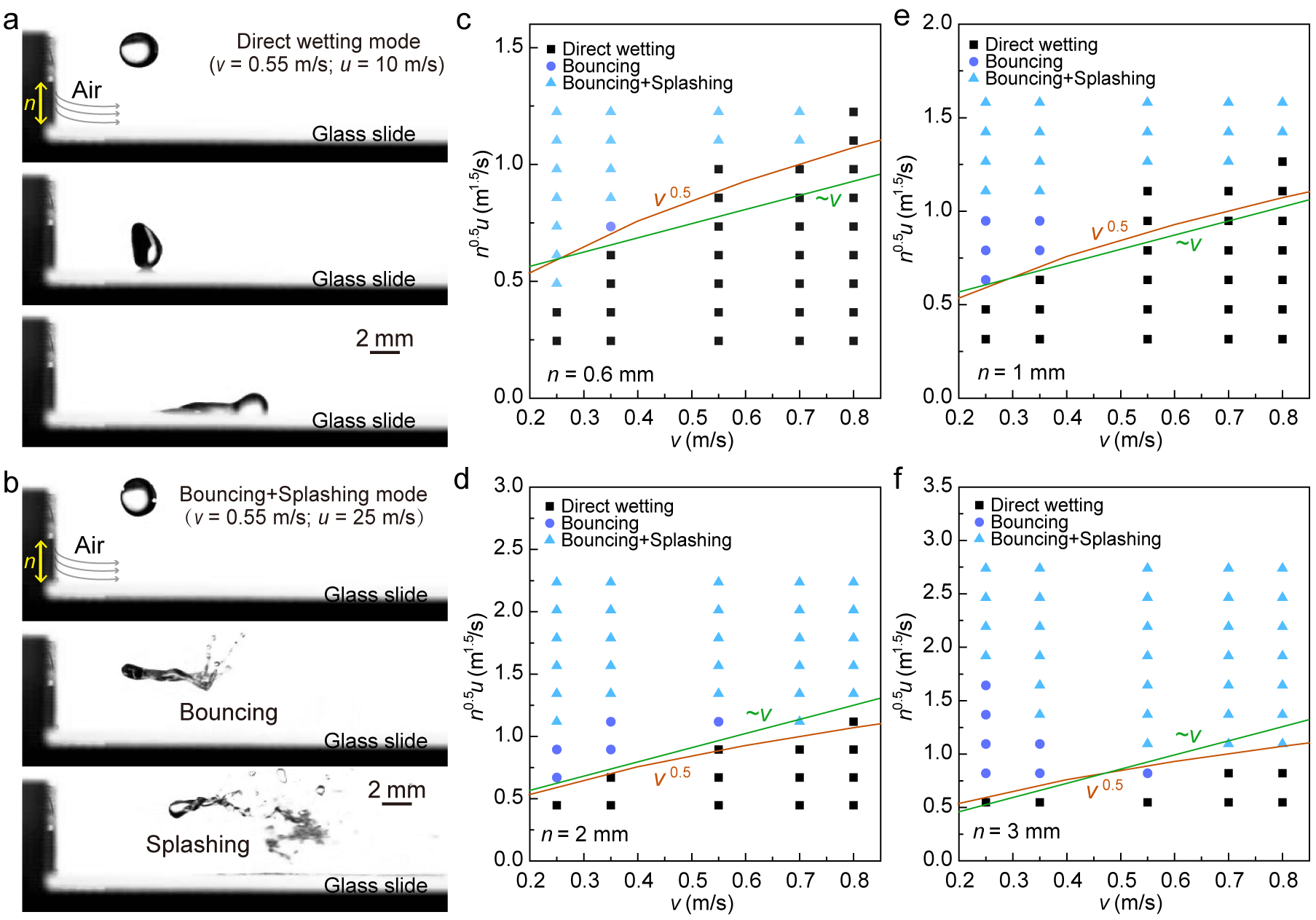

Supplement: Supplementary 2 — Movie S1. Drop impacting on an air layer surface: Bouncing mode. Movie S2. Drop wetting on a smooth surface without an air layer. Movie S3. Drop impacting on an air layer surface: Direct wetting mode. Movie S4. Drop impacting on an air layer surface: Bouncing and splashing mode. Movie S5. Drop bouncing on an inclined air layer surface. [file research.0111.f2.zip › FigureS3.tif]
